# Inflammatory Markers in Anorexia Nervosa: An Exploratory Study

**DOI:** 10.3390/nu10111573

**Published:** 2018-10-24

**Authors:** Bethan Dalton, Iain C. Campbell, Raymond Chung, Gerome Breen, Ulrike Schmidt, Hubertus Himmerich

**Affiliations:** 1 Section of Eating Disorders, Department of Psychological Medicine, Institute of Psychiatry, Psychology & Neuroscience, King’s College London, London SE5 8AF, UK; iain.campbell@kcl.ac.uk (I.C.C.); ulrike.schmidt@kcl.ac.uk (U.S.); hubertus.himmerich@kcl.ac.uk (H.H.); 2 MRC Social, Genetic, and Developmental Psychiatry Centre, Institute of Psychiatry, Psychology & Neuroscience, King’s College London, London SE5 8AF, UK; raymond.chung@kcl.ac.uk (R.C.); gerome.breen@kcl.ac.uk (G.B.); 3 National Institute for Health Research Biomedical Research Centre for Mental Health, Institute of Psychiatry, Psychology & Neuroscience at the Maudsley Hospital and King’s College London, London SE5 8AF, UK; 4 South London and Maudsley NHS Foundation Trust, Bethlem Royal Hospital, Monks Orchard Road, Beckenham, Kent BR3 3BX, UK

**Keywords:** anorexia nervosa, inflammatory markers, inflammation, cytokines, chemokines, adhesion molecules

## Abstract

Inflammation has been suggested to play a pathophysiological role in anorexia nervosa (AN). In this exploratory cross-sectional study, we measured serum concentrations of 40 inflammatory markers (including cytokines, chemokines, and adhesion molecules) and brain-derived neurotrophic factor (BDNF) in people with AN (*n* = 27) and healthy controls (HCs) (*n* = 13). Many of these inflammatory markers had not been previously quantified in people with AN. Eating disorder (ED) and general psychopathology symptoms were assessed. Body mass index (BMI) and body composition data were obtained. Interleukin (IL)-6, IL-15, and vascular cell adhesion molecule (VCAM)-1 concentrations were significantly elevated and concentrations of BDNF, tumor necrosis factor (TNF)-β, and vascular endothelial growth factor (VEGF)-A were significantly lower in AN participants compared to HCs. Age, BMI, and percentage body fat mass were identified as potential confounding variables for several of these inflammatory markers. Of particular interest is that most of the quantified markers were unchanged in people with AN, despite them being severely underweight with evident body fat loss, and having clinically significant ED symptoms and severe depression and anxiety symptoms. Future research should examine the replicability of our findings and consider the effect of additional potential confounding variables, such as smoking and physical activity, on the relationship between AN and inflammation.

## 1. Introduction

Anorexia nervosa (AN) is a serious psychiatric disorder characterised by low body weight due to food restriction and weight-control behaviours, such as excessive exercise and self-induced vomiting, together with an intense fear of weight gain and disturbed body perception [1]. Altered concentrations of inflammatory markers, in particular cytokines, have been reported in people with AN [2,3]. Cytokines are cell signalling molecules produced by a range of cells (e.g., microglia, astrocytes) in the brain and the periphery (e.g., by macrophages and T-lymphocytes) and are essential in coordinating responses to infection [4]. In addition, changes in the circulating concentrations and production of cytokines have been associated with a range of disease states, including obesity [5] and diabetes [6], as well as depression [7], schizophrenia [8], and eating disorders (EDs) [2,3].

Research in AN has primarily focused on pro-inflammatory cytokines, which promote and up-regulate inflammatory reactions [4]. Recent meta-analyses have concluded that the pro-inflammatory cytokines tumor necrosis factor (TNF)-α and interleukin (IL)-6, are elevated in people with AN, compared to healthy individuals (for reviews see: [2,3]). However, few studies have quantified the concentrations of cytokines in other categories, such as T-helper (T_H_)-1, T_H_2, and anti-inflammatory cytokines (e.g., IL-10), the latter of which play an immunomodulatory role by reducing inflammation [9]. An example of one such cytokine yet to be measured in people with AN is TNF-β, which is produced by T_H_1 cells. TNF-β performs a variety of important roles in immune regulation [10,11], but has also been implicated in the regulation of the commensal gut microbiota [12,13,14], which appears to be involved in the pathology of AN [15,16,17]. Additionally, a number of cytokines implicated in other disorders, such as depression and obesity, are yet to be measured in AN. One example is IL-17, a T_H_17 cytokine that has been reported to predict treatment response in people with depression [18], and seems to be involved in the pathophysiology of schizophrenia [19] and the molecular and cellular effects of antipsychotics [20].

Chemokines are a subcategory of smaller cytokines known to induce chemotaxis, with some also having a homeostatic function in relation to haematopoiesis, immune surveillance, and adaptive immune system responses [21,22]. The chemokines RANTES, monocyte chemoattractant protein (MCP)-1, and fractalkine have been measured in two studies in people with AN [23,24]. Similarly, adhesion molecules, which mediate the binding of cells in the immune system [25], have been measured in one study in a sample of people with AN [26]. Circulating concentrations of vascular cell adhesion molecule (VCAM)-1 have been reported to be elevated in people with AN compared to healthy participants, but intercellular adhesion molecule (ICAM)-1 did not differ between the groups.

Cytokines and chemokines impact several biological domains implicated in the pathophysiology of AN, including the modulation of neurotransmitter systems, neuroendocrine functioning, and neural plasticity [27,28,29,30,31]. For example, in the depression literature, it has been hypothesised that elevated pro-inflammatory cytokine levels may lead to symptoms of depression, partly via their disruption of growth factor production, e.g., brain-derived neurotropic factor (BDNF) [32] and vascular endothelial growth factor (VEGF)-A [33], which has a subsequent effect on adult neurogenesis [28,34]. Disruption to these biological processes can then lead to alterations in mental state, including affect, learning and memory, and behaviour (e.g., depressive-like behaviours) [28,35].

A number of factors, including body mass index (BMI), age, medication, and smoking status, have been reported to influence cytokine concentrations [36]. These may be potential confounding factors in studies of the role of cytokines in AN, particularly given the low weight seen in AN, the tendency for research in EDs to focus on adolescents and young adults [2], and research indicating that people with EDs report higher rates of smoking than healthy controls [37]. Previous studies have not assessed the potential impact of depression symptoms on cytokine concentrations in AN. A pro-inflammatory profile has been identified in people with depression [38] and the comorbidity between AN and unipolar depression is of significant clinical relevance, as approximately 40% of people receiving treatment for AN also suffer from depression [39]. Therefore, it is unclear as to whether the alterations observed in cytokine concentrations are due to the AN or symptoms of comorbid disorders, such as depression.

Few studies have considered a broad range of cytokines and other markers involved in inflammatory processes and their potential role in the biological profile of AN. Therefore, in this exploratory cross-sectional study, we measured a variety of inflammatory markers in a sample of AN participants and healthy controls (HCs) to determine whether these markers are altered in AN. Several of these inflammatory markers have not been previously quantified in people with AN. A secondary objective was to test for the effects of potential confounders on concentrations of the inflammatory markers, including age and BMI, and explore the effect of current symptom severity on markers of inflammation in people with AN.

## 2. Materials and Methods

### 2.1. Participants and Study Design

Between 2010 and 2013, 55 participants with AN (outpatients *n* = 27; day-patients *n* = 10; inpatients *n* = 18) and 30 HCs were recruited as part of the “Relationship between Overactivity, Stress and Anxiety in Anorexia Nervosa” (ROSANA) study [40,41]. Female adults with a primary diagnosis of AN (restricting or binge-eating/purging type) and a BMI < 17.5 kg/m^2^ were recruited within the first four weeks of treatment for AN via Specialist Eating Disorder Services in and around London. HCs (*n* = 30) were recruited via an e-mail circular to students and staff at King’s College London. Exclusion criteria for HCs were a history of or current mental health disorder, including EDs, and the presence of physical illness, which were assessed in the initial research session using a specially designed record form and the research version of the Structured Clinical Interview for Diagnostic and Statistical Manual of Mental Disorders (DSM)-IV Axis I Disorders [42], described below. All participants provided informed consent before study participation. The study was conducted in accordance with the Declaration of Helsinki and the study received ethical approval from the South East London Research Ethics Committee (REC ref: 09/H0807/4).

### 2.2. Measures

#### 2.2.1. Demographic Characteristics and Screening

A specially designed record form was used to collect demographic data and additional information relating to the inclusion and exclusion criteria described above, e.g., the presence of medical conditions. To determine smoking status, participants were asked if they smoked and if so, to report the number of cigarettes smoked per day. For AN participants, illness duration (in this case, time since diagnosis) was also recorded. All participants completed the research version of the Structured Clinical Interview for DSM-IV Axis I Disorders [42], a validated structured interview, to confirm diagnosis in the AN participants and identify a history of and/or current mental health problems in the HCs.

#### 2.2.2. Anthropometry

Height and body weight were measured, and from these measurements, BMI (kg/m^2^) was calculated. Body composition was also measured using a portable and non-invasive Inbody S10 machine (Inbody Co., Ltd., Seoul, Korea) which uses the Bioelectrical Impedance Analysis (BIA) measurement method. Following the input of height and weight details, this machine provides data on muscle and fat, bone mineral content, intracellular and extracellular water, protein, and minerals. The calculations used to do this are based on the assumption that the body is a cylindrical-shaped conductor. Resistance is low in lean tissue (as it contains the majority of intracellular and extracellular fluid and is thus a good conductor of electrical current), and fat mass is high in resistance as it is does not contain any water (and thus does not conduct electrical current). Based on the assumption that impedance (resistance) is proportional to total body water, predictive equations then determine total body water, total body fat, and lean tissue mass. Given that adipose tissue has been implicated in the genesis of cytokines and produces certain pro-inflammatory cytokines (e.g., IL-6), we focused on the association between inflammatory markers and body fat percentage and did not include other body composition parameters in our analyses.

#### 2.2.3. Eating Disorder Behaviours and General Psychopathology

ED symptoms were assessed using the Eating Disorder Examination-Questionnaire (EDE-Q) [43]. This questionnaire has 36-items assessing ED symptoms and behaviours over the previous 28 days. A global score can be calculated, and items can also be categorised and scored into the following four subscales: restraint, eating concern, weight concern, and shape concern. Related psychopathology was assessed using the Depression Anxiety Stress Scale 21-Version (DASS-21) [44]. This is a 21-item questionnaire measuring symptoms of general psychopathology over the previous seven days. As well as a total score, a score for the three subscales—depression, anxiety, and stress—can be calculated.

Additional measures related to physical activity were also collected and findings are reported elsewhere [40,41].

#### 2.2.4. Inflammatory Markers

Blood samples were collected, and serum was stored at −80 °C prior to use. Serum was thawed at room temperature and the concentrations of 42 inflammatory markers were quantified simultaneously using multiplex ELISA-based technology provided by the Meso Scale Discovery V-PLEX Human Biomarker 40-Plex Kit and a customised human duplex kit assaying BDNF and interferon (IFN)-α, following the manufacturer’s instructions (Meso Scale Diagnostics, LLC., Rockville, MD, USA). Cases and controls were randomised across batches, and plates were scanned on the Mesoscale Scale Discovery MESO Quickplex SQ 120 reader at the MRC SGDP Centre, Institute of Psychiatry, Psychology & Neuroscience, King’s College London. The inflammatory markers measured in the 40-plex array were: basic fibroblast growth factor (bFGF), C-reactive protein (CRP), Eotaxin, Eotaxin-3, Fms-like tyrosine kinase (Flt)-1, granulocyte-macrophage colony-stimulating factor (GM-CSF), ICAM-1, IFN-γ, IL-1α, IL-1β, IL-2, IL-4, IL-5, IL-6, IL-7, IL-8, IL-10, IL-12/IL-23p40, IL-12p70, IL-13, IL-15, IL-16, IL-17A, interferon γ-induced protein (IP)-10, MCP-1, MCP-4, macrophage inflammatory protein (MIP)-1α, MIP-1β, placental growth factor (PlGF), serum amyloid A (SAA), thymus and activation-regulated chemokine (TARC), tyrosine kinase (Tie)-2, TNF-α, TNF-β, VCAM-1, VEGF-A, VEGF-C, and VEGF-D. The assay for macrophage-derived chemokine (MDC) was not included in the current panel due to quality control issues.

### 2.3. Statistical Analysis

Thirty-two AN and 14 HC participants had available blood samples. One HC participant was excluded from the analyses due to having a BMI below 18.5 kg/m^2^, i.e., in the underweight range. Five AN participants were excluded from analyses due to reported autoimmune and/or inflammatory diseases. Therefore, the current cross-sectional analyses are based on a sample consisting of 27 participants with a diagnosis of AN and 13 HCs.

Standard curves were used to determine absolute quantities (pg/mL) of each inflammatory marker. All statistical analyses were performed in Stata 15 [45].

#### 2.3.1. Cross-Sectional Comparisons

For demographics and clinical characteristics, group comparisons were assessed using t-tests or Mann-Whitney U-tests depending on the distribution of the data. Log-transformation of inflammatory marker values was conducted due to the presence of outliers and non-normal distributions; however, data remained non-normal for most of the measured molecules. Therefore, Mann-Whitney U-tests were employed to compare concentrations of inflammatory markers between the AN and HC groups. IFN-α was only detected in three samples (two HC and one AN); the results are therefore not presented.

#### 2.3.2. Exploratory Regression Analyses

Absolute quantities of the inflammatory markers (pg/mL) were log-transformed to allow for regression analyses. To identify marker-specific confounders, linear regressions were performed for each log-transformed inflammatory marker (as the dependent variable) with each potential confounding variable (age, BMI, percentage fat mass) separately in the whole sample. To test the effect of illness severity on inflammatory marker concentrations, we performed linear regressions in the AN participants, with the log-transformed inflammatory marker as the dependent variable and illness duration or ED symptoms, as measured by the EDE-Q, as the independent variable. To test the effect of general psychopathology on inflammatory marker concentrations, linear regressions in the AN participants, with the log-transformed inflammatory marker as the dependent variable and the total DASS-21 score as the independent variable, were conducted. For both sets of analyses, studentized residuals greater than ±3 standard deviations were deemed to be outliers and were removed, and assumptions were tested and met.

The level of significance was set at *p* < 0.05, and as this was an exploratory study, levels of significance were not adjusted for multiple testing.

## 3. Results

Demographic, anthropometric, and clinical characteristics of the AN participants and HCs are presented in Table 1. All participants were female. Mean age did not significantly differ between the AN and HC groups (*U* = 144*, z* = −1.36, *p* = 0.1735). Seven participants with AN reported being a current smoker, with an average of 9.14 ± 5.90 cigarettes smoked per day. As expected, AN participants had lower BMI (*t* (38) = 7.88, *p* < 0.001) and percentage body fat (*U* = −22*, z* = 3.63, *p* = 0.0003) scores, and higher EDE-Q scores (global score: *U* = 85.5*, z* = −4.87, *p* < 0.001) than HCs. The EDE-Q global score for the AN participants was greater than the commonly used clinical cut-off score of 4 e.g., [46,47]. AN participants also reported greater depression, anxiety, and stress than HCs on the DASS-21 (total score: *U* = 92.5*, z* = −4.67, *p* <0.001). Proposed cut-off scores [44] suggest that the level of severity that AN participants reported was severe for depression, anxiety, and stress.

### 3.1. Inflammatory Markers

#### 3.1.1. Cross-Sectional Comparison

The median concentrations (pg/mL) of the quantified inflammatory markers, along with the interquartile range, and minimum and maximum values, for HCs and AN participants are shown in Table 2. Median serum levels of TNF-β (*U* = 76, *z* = 2.87, *p* = 0.004) and VEGF-A (*U* = 102, *z* = 2.12, *p* = 0.030) were lower in AN participants than in HCs. Median concentrations of IL-6 (*U* = 92, *z* = −2.41, *p* = 0.016), IL-15 (*U* = 85, *z* = −2.61, *p* = 0.009), and VCAM-1 (*U* = 106, *z* = −2.01, *p* = 0.032) were found to be higher in AN participants compared to HCs. No other inflammatory parameters were found to differ between groups.

#### 3.1.2. Identification of Marker-Specific Confounding Variables

The full findings of the regressions assessing age, BMI, and percentage fat mass as potential confounders of the serum concentrations of inflammatory markers across all participants are shown in Table S1. Age significantly influenced the serum concentrations of Eotaxin-3 (*F*(1, 37) = 5.68, *p* = 0.0224), IFN-γ (*F*(1, 33) = 7.91, *p* = 0.0082), MCP-1 (*F*(1, 38) = 8.87, *p* = 0.0050), MIP1-α (*F*(1, 38) = 4.48, *p* = 0.0408), SAA (*F*(1, 38) = 6.99, *p* = 0.0119), and TNF-α (*F*(1, 38) = 5.07, *p* = 0.0302). BMI significantly predicted concentrations of IL-4 (*F*(1, 34) = 4.73, *p* = 0.0367), IL-6 (*F*(1, 38) = 9.48, *p* = 0.0039), IL-10 (*F*(1, 37) = 6.67, *p* = 0.0139), IL-12/IL-23p40 (*F*(1, 38) = 10.97, *p* = 0.0020), IL-15 (*F*(1, 38) = 9.54, *p* = 0.0037), TNF-β (*F*(1, 35) = 7.45, *p* = 0.0098), and VEGF-C (*F*(1, 37) = 6.17, *p* = 0.0177). Percentage fat mass significantly influenced IL-6 (*F*(1, 38) = 8.83, *p* = 0.0052), IL-12/IL-23p40 (*F*(1, 37) = 10.05, *p* = 0.0031), TNF-β (*F*(1, 35) = 4.93, *p* = 0.0331), and VEGF-C (*F*(1, 38) = 5.00, *p* = 0.0315). Age, BMI, and percentage fat mass were not found to be associated with concentrations of any of the remaining inflammatory markers.

#### 3.1.3. Effect of Illness Severity

Full results from the regressions determining the effect of illness severity, in relation to illness duration, ED symptoms, and general psychopathology, on serum concentrations of the inflammatory markers in AN participants only are shown in Table S2. Illness duration of AN participants significantly predicted serum concentrations of IL-4 (*F*(1, 18) = 15.82, *p* = 0.0009), IL-12/IL-23p40 (*F*(1, 21) = 6.70, *p* = 0.0172), MCP-1 (*F*(1, 21) = 6.40, *p* = 0.0194), and VEGF-A (*F*(1, 22) = 5.55, *p* = 0.0278). Severity of ED symptoms in AN participants, as measured by the EDE-Q, predicted serum concentrations of IP-10 (*F*(1, 23) = 12.60, *p* = 0.0017) and PlGF (*F*(1, 25) = 4.44, *p* = 0.0454). Level of general psychopathology (symptoms of depression, anxiety, and stress, as measured by the DASS-21) significantly predicted Eotaxin (*F*(1, 25) = 4.64, *p* = 0.0410), IL-7 (*F*(1, 25) = 4.60, *p* = 0.0419), IL-8 (*F*(1, 25) = 10.08, *p* = 0.0040), IP-10 (*F*(1, 25) = 6.06, *p* = 0.0211), MCP-1 (*F*(1, 25) = 5.43, *p* = 0.0282), and TARC (*F*(1, 22) = 9.18, *p* = 0.0062) concentrations. No other inflammatory markers were significantly associated with illness duration or severity of ED and general psychopathology symptoms. 

### 3.2. Brain-Derived Neurotrophic Factor

Median serum levels of BDNF were reduced in AN participants compared to HCs (*U* = 101, *z* = 2.15, *p* = 0.0320). Concentrations of BDNF were significantly influenced by BMI (*F*(1, 38) = 13.86, *p* = 0.0007) and percentage body fat mass (*F*(1, 38) = 7.08, *p* = 0.0114), but not by age, as shown in Table S1. Illness duration, ED symptom severity, and general psychopathology symptoms were not associated with serum concentrations of BDNF (see Table S2).

## 4. Discussion

### 4.1. Summary of Findings

We measured a range of markers involved in inflammatory processes, including cytokines, chemokines, acute-phase reactants, and cell adhesion molecules, in people with AN and HCs. Median concentrations of BDNF, IL-6, IL-15, TNF-β, VCAM-1, and VEGF-A were found to differ between AN and HCs, with IL-6, IL-15, and VCAM-1 being elevated in AN, and BDNF, TNF-β, and VEGF-A being reduced, compared to HCs. No other inflammatory markers differed between AN participants and HCs. Our exploratory analyses also identified potential confounding variables of these markers, including age, BMI, and percentage fat mass, which should be considered in future studies. Illness severity, both with respect to ED and general psychopathology symptoms, predicted concentrations of certain inflammatory markers that were not found to differ between AN participants and HCs. Of the markers with significant cross-sectional differences, illness duration predicted serum concentrations of VEGF-A.

#### 4.1.1. Inflammatory Markers

Given that the AN patients in this sample are seriously unwell, as evidenced by their low BMI, the loss of fat mass, and the presence of clinically significant ED and severe depression and anxiety symptoms, the finding that most inflammatory markers (*n* = 33) were unchanged may seem somewhat surprising. However, it has been proposed that the functioning of the immune system is relatively preserved in AN, despite malnutrition [48]. For example, extensive evidence suggests that patients with AN tend to be free from infectious diseases, and rarely have colds and/or flu (at least until very advanced stages of the disease) [48,49], which is in contrast to the heightened risk of infection observed in those with typical malnutrition. A recent in vitro study of immune parameters in people with AN reported that despite reductions in some immune cell populations, AN is also associated with enhanced antioxidant potential and anti-inflammatory status, in comparison to HCs [50]. This may contribute to the preservation of immune system functioning reported in AN. However, it needs to be considered that data derived from in vitro methods may not reflect how cells would respond in vivo. An additional contributing factor could be a relatively sustained/unaffected gut barrier function in AN [51], in spite of the distinct alterations of the gut microbiome and low gut microbiome diversity reported in AN patients [15]. A ‘leaky gut’ is associated with low-grade inflammation (i.e., leaking of bacteria and/or their components from the gut into circulation is thought to elicit an inflammatory response) [52,53]; therefore, sustained gut permeability will reduce the likelihood of an inflammatory response.

To the best of our knowledge, this is the first time serum concentrations of IL-15, TNF-β, and VEGF-A have been measured and were found to be altered in AN patients. Therefore, we have discussed the potential significance of these findings in more detail. 

IL-15, a T cell growth factor, has been suggested to be involved in the modulation of serotonergic transmission [54,55], which may underlie the depressive symptoms and sleep disturbances that are often present in people with AN, and fits with existing evidence of serotonergic alterations in AN [56,57]. Elevations of IL-15 levels have also been reported in patients with schizophrenia [58], which is interesting in light of the possible genetic overlaps between AN and schizophrenia that have been identified in genome-wide association study (GWAS) data [59]. IL-15 has also been implicated in cross-talk between fat and muscle and reported to have an anabolic role, i.e., decreasing fat and increasing muscle mass [60]. It is unclear, therefore, why it is increased in the anorectic group given that they show a severe loss of body fat. It may, for example, be raised as a way of trying to maintain muscle mass in the ill state.

TNF-β (also known as lymphotoxin-α) has several roles in immune regulation [10] and is involved in the regulation of cell survival, proliferation, differentiation, and apoptosis [11]. A TNF-β polymorphism (+252G/A) has been proposed to increase the risk of developing schizophrenia [61]. TNF-β also has a role in maintaining lipid homeostasis, which is potentially important in AN. However, what is perhaps of most interest is that it is involved in regulating intestinal microbiota [12,13,14], especially as the gut microbiome has been implicated in the pathophysiology of AN e.g., [15].

VEGF-A induces angiogenesis, vasculogenesis, and endothelial cell growth, and also influences vascular permeability, similar to VCAM-1 [62]. Altered levels of VEGF-A have also been found in patients with depression [63] and schizophrenia [64]. Lastly, VEGF-A has been suggested to enhance adult neurogenesis and hippocampus-dependent learning and memory [33], which may be important in both responsivity to illness and in relation to therapy. 

Taken together, changes in IL-15, TNF-β, and VEGF-A could potentially contribute to the development of AN symptoms, such as low mood and disturbed sleep, as well as its clinical consequences, such as impaired learning and memory. Furthermore, they provide a link between the biological pathophysiology of AN with depression and schizophrenia, which are clinically- and genetically-related psychiatric disorders. This association may be suggestive of inflammatory markers being indicators of transdiagnostic symptoms, such as low mood, rather than specific psychiatric diagnoses. Also, such a biological association between these disorders could have clinical and therapeutic relevance. For example, we could consider the question as to whether antipsychotics, such as olanzapine, which is approved for the treatment of schizophrenia and alters cytokine production [65], might help patients with AN [31,66]. This may also be of particular interest in AN as olanzapine has been shown to alter the gut microbiome, which could additionally contribute to weight gain [67,68,69,70].

The findings of increased IL-6 and VCAM-1 serum concentrations in our sample of people with AN compared to HCs are of less novelty, but indicate the reliability of our findings, as these results have also been reported previously.

As described, there are already a number of studies that have consistently identified an association between AN and the pro-inflammatory cytokine IL-6 [2]. IL-6 is an inducer of the acute-phase response, which has been shown to have suppressive effects on food intake [71] and inhibit adipogenesis [72]. Our results replicated the findings of increased concentrations of IL-6 in people with AN, as compared to HCs.

Víctor et al. [26] previously reported increased VCAM-1 serum levels in patients with AN. VCAM-1 is a cell adhesion molecule with a key role in leukocyte recruitment from blood into tissue and is thus important for cellular immune response [73]. Because of its wide distribution in human tissues and organs, VCAM-1 has been implicated in the development of a variety of pathophysiological states in the brain and in the body periphery, including autoimmune diseases, cardiovascular disease, and infections [74].

It is unclear why these particular inflammatory markers are altered in people with AN compared to HCs. However, there are a number of potential factors which may contribute to these alterations, including stress and neuroendocrine functioning, genetics, the gut microbiota, early life stress, and negative health behaviours (e.g., disturbed sleep, altered diet, smoking) [75]. 

#### 4.1.2. Brain-Derived Neurotrophic Factor

The reduced serum concentrations of BDNF in AN participants compared to HCs is consistent with previous findings [76]. BDNF is a neurotrophin implicated in both central and peripheral nervous system development. It is well-established that BDNF and cytokines cross-regulate each other. Certain pro-inflammatory cytokines can suppress the expression of BDNF [34] and BDNF-dependent synaptic plasticity [32]. It is thought that the detrimental effect of pro-inflammatory cytokines on neuroplasticity may be mediated by BDNF [34]. Taking together the evidence in AN of elevated pro-inflammatory cytokines [2,3], reduced concentrations of BDNF and VEGF-A [76], and reduced hippocampal volumes [77,78], a key area for adult neurogenesis, it could be hypothesised that this mechanism may be at play in AN, as proposed in the depression literature [34,79]. This hypothesis would be consistent with the high prevalence (approximately 40%) of depression in patients with AN [39].

#### 4.1.3. Marker-Specific Confounding Variables

Our exploratory regression analyses found that BMI significantly predicted BDNF, IL-4, IL-6, IL-10, IL-12/IL-23p40, IL-15, TNF-β, and VEGF-C concentrations. BDNF, IL-6, IL-12/IL-23p40, TNF-β, and VEGF-C were also significantly predicted by percentage fat mass. Previous studies assessing IL-6 in AN have failed to control for BMI in cross-sectional analyses. Therefore, we cannot be certain that the alterations we identified in concentrations of BDNF, IL-6, IL-15, and TNF-β are attributable to AN, rather than simply BMI or percentage fat mass. We also identified age as a significant confounder of Eotaxin-3, IFN-γ, MCP-1, MIP1-α, SAA, and TNF-α. Therefore, studies in larger samples with adequate power should ensure that BMI, and other confounding variables, such as age and percentage fat mass, are incorporated into analyses as covariates to further explore this relationship. Research is also needed to consider the effect of additional potential confounding variables on the relationship between AN and inflammation. Physical activity, for example, may be particularly pertinent in AN patients, given that excessive exercise is often a key feature of the disorder.

### 4.2. Strengths and Limitations

This study is the first to measure several inflammatory markers in patients with AN, including IL-15 and TNF-β, identifying several alterations in inflammatory markers in AN that warrant future research in larger samples. We also assessed illness severity, in terms of illness duration; ED symptoms, including psychological and anthropometric measures; and associated psychopathology (e.g., depression and anxiety), in our participants. Few previous studies have included such variables [2], which in the current study have allowed us to explore the relationship between illness severity and inflammatory markers. In addition, for the HCs, the median values of a number of the assessed markers are similar to those observed in previous studies, suggesting that our HC group is a valid comparison group.

Several limitations should be noted. The sample is small, which limits the power in this study and due to the exploratory nature, we did not correct for multiple testing, thus increasing the likelihood of receiving a significant result. Additionally, while the AN and HC groups did not differ in mean age, it must be mentioned that the AN group had a larger age range (18–67 years) than the HCs (20–36 years). The AN sample included both inpatients and outpatients; differences between these treatment settings in the opportunity to engage in ED behaviours, such as calorie restriction, self-induced vomiting, and excessive exercise, may effect cytokine concentrations e.g., [80,81]. As BIA measurements of body composition are influenced by fluid and electrolyte status (for which there are known imbalances in AN and these parameters are reported to be particularly affected in early refeeding), the accuracy of BIA measurements of body composition may be limited, particularly in the AN group [82,83]. For practical reasons, it was not possible to ensure blood samples were drawn at a specific time of day and as cytokine production and release is reported to occur in a circadian manner, it is possible that some natural variations may have occurred [84]. As previously described, the inflammatory markers measured in the current study can be affected by a number of pre-analytical factors [36], including age, BMI, smoking, and medication. We did not have data on medication for all participants; however, research has shown that antidepressant and antipsychotic medication can influence cytokine concentrations and production [20,85]. As these medications are prescribed to AN patients to target comorbid features of AN, such as depression and/or to induce weight gain [31], it would be important to identify medication status and incorporate this into analyses as a potential covariate. However, the limited power in this study precludes more complex analyses, such as incorporating several confounding variables or considering the effect of nominal confounders, such as smoking status, on inflammatory markers. Overall, future investigations of inflammatory markers in AN need to ensure that such confounders are assessed and reported, and if possible, accounted for in statistical analyses.

## 5. Conclusions

This exploratory study measured a broad range of inflammatory markers, many of which had not been previously assessed in AN. IL-15, VEGF-A, and TNF-β, for the first time, were shown to be altered in people with AN in comparison to HCs. Previous findings regarding an elevation of IL-6 and VCAM-1 and a reduction in BDNF in AN participants were replicated. We also considered age, BMI, and percentage fat mass as potential confounding variables of concentrations of the inflammatory markers. Our findings suggest that future research should include covariates in analyses of this relationship to explore whether this may account for some of the group differences in inflammatory markers observed in the current study. Finally, given that these inflammatory markers function as part of a complex network, future studies in larger samples should consider developing a composite score of cytokine concentrations. 

## Figures and Tables

**Table 1:** Demographic, anthropometric, and clinical characteristics for AN participants and HCs.

Demographic, Anthropometric, and Clinical Characteristics	Healthy Controls (*n* = 13)	Anorexia Nervosa *(n* = 27)	*p* Value
**Demographics**			
Age (years) (mean ± SD)	25.54 ± 4.52	31.48 ± 11.40	0.1735
Current smoker (*n*)	0 ^a^	7	
**Anthropometrics**			
BMI (kg/m^2^) (mean ± SD)	20.88 ± 1.68	15.33 ± 2.25	<0.0010
Body fat (%) (mean ± SD)	17.08 ± 6.05 ^b^	7.76 ± 6.07	0.0003
**Clinical characteristics**			
Duration of diagnosis, years (mean ± SD)		11.64 ± 11.54 ^c^	
AN-R/AN-BP (*n*)		12/15	
Current outpatient/inpatient (*n*)		16/11	
EDE-Q Global (mean ± SD)	0.66 ± 0.70	4.20 ± 1.27	<0.0010
EDE-Q Restraint (mean ± SD)	0.62 ± 0.88	4.04 ± 1.77	<0.0010
EDE-Q Eating Concern (mean ± SD)	0.26 ± 0.48	3.82 ± 1.28	<0.0010
EDE-Q Weight Concern (mean ± SD)	0.74 ± 0.83	4.13 ± 1.59	<0.0010
EDE-Q Shape Concern (mean ± SD)	1.02 ± 0.85	4.82 ± 1.24	<0.0010
DASS-21 Total (mean ± SD)	13.85 ± 13.89	72.30 ± 33.32	<0.0010
DASS-21 Depression (mean ± SD)	3.54 ± 5.43	24.59 ± 13.73	<0.0010
DASS-21 Anxiety (mean ± SD)	3.08 ± 4.94	19.48 ± 11.84	0.0001
DASS-21 Stress (mean ± SD)	7.23 ± 5.20	28.22 ± 10.69	<0.0010

^a^*n* = 6 missing; ^b^ *n* = 1 missing; ^c^ *n* = 3 missing. Abbreviations: HCs—healthy controls; AN—anorexia nervosa; SD—standard deviation; BMI—body mass index; AN-R—anorexia nervosa restricting type; AN-BP—anorexia nervosa binge-eating/purging type; EDE-Q—Eating Disorder Examination—Questionnaire; DASS-21—Depression Anxiety and Stress Scales-21 Version.

**Table 2:** Median serum concentrations (pg/mL), with interquartile range (IQR), minimum and maximum values, of inflammatory markers for HCs and AN participants.

Inflammatory Marker	Healthy Controls					Anorexia Nervosa					*p* Value
	*N*	Median	IQR	Min	Max	N	Median	IQR	Min	Max	
**a. Concentrations significantly lower in AN compared to HCs**											
BDNF	13	17,375.24	7862.72	10,526.40	21,884.09	27	12,799.75	5947.87	7792.55	23,003.72	0.0315
TNF-β	13	0.86	0.32	0.43	53.22	27	0.60	0.19	0.16	1.22	0.0041
VEGF-A	13	471.96	220.76	205.98	749.41	27	288.82	155.41	133.70	973.73	0.0338
**b. Concentrations significantly higher in AN compared to HCs**											
IL-6	13	0.38	0.29	0.10	1.11	27	0.49	0.90	0.20	3.35	0.0159
IL-15	13	2.54	0.39	1.91	3.18	27	2.90	0.81	1.57	5.24	0.0090
VCAM-1	13	612,378.30	92,337.87	402,297.20	739,682.80	27	709,059.60	220,832.10	434,057.50	1,069,997.00	0.0448
**c. Concentrations not significantly different between groups**											
bFGF	13	11.01	10.73	2.17	21.13	27	12.13	13.39	1.15	65.25	0.3334
CRP	13	332,422.10	1,497,240.00	38,821.00	4,878,443.00	27	342,975.80	5,408,764.00	39,716.48	37,500,000.00	0.9424
Eotaxin	13	208.55	61.81	136.51	285.80	27	175.47	123.38	101.36	511.08	0.8511
Eotaxin-3	13	20.55	6.48	9.95	112.91	27	15.15	14.22	5.14	60.87	0.1058
Flt-1	13	61.83	11.64	27.93	86.21	27	68.69	28.37	11.99	117.06	0.1224
GM-CSF	13	0.15	0.21	0.02	0.88	22	0.19	0.17	0.01	0.35	0.8779
ICAM-1	13	658,988.00	183,427.10	517,283.10	861,717.20	27	701,224.80	244,044.70	415,274.60	977,044.00	0.1224
IFN-γ	13	3.94	1.64	2.11	6.84	27	4.64	5.72	2.11	64.28	0.1448
IL-1α	13	1.13	1.32	0.41	3.51	27	1.01	0.64	0.44	6.00	0.4105
IL-1β	9	0.20	0.33	0.04	1.86	21	0.19	0.19	0.01	4.07	0.7343
IL-2	6	0.12	0.24	0.03	0.43	13	0.15	0.16	0.01	0.85	0.9301
IL-4	13	0.08	0.07	0.01	0.20	25	0.05	0.05	0.00	5.24	0.1481
IL-5	12	1.09	0.87	0.43	3.45	24	1.29	1.01	0.07	4.39	0.7626
IL-7	13	13.66	6.11	8.76	20.13	27	12.58	6.80	5.82	26.20	0.8966
IL-8	13	33.51	62.10	8.44	503.23	27	23.09	92.94	5.80	388.41	0.4271
IL-10	13	0.18	0.10	0.06	0.41	27	0.28	0.26	0.04	4.40	0.3120
IL-12/IL-23p40	13	117.69	49.83	72.15	250.50	27	92.00	50.59	38.72	220.89	0.0806
IL-12p70	11	0.18	0.09	0.01	0.53	25	0.19	0.25	0.01	1.06	0.7572
IL-13	9	2.95	3.25	1.53	10.34	17	2.44	3.13	0.76	6.35	0.2692
IL-16	13	160.52	58.89	99.73	291.95	27	183.59	194.22	127.93	463.24	0.1702
IL-17A	13	1.78	1.94	0.36	3.85	27	1.91	1.51	0.63	43.83	0.9539
IP-10	13	110.93	57.70	75.89	175.65	27	115.99	108.88	72.71	596.43	0.3191
MCP-1	13	208.55	126.78	140.94	406.88	27	191.53	77.31	121.51	345.28	0.3480
MCP-4	13	142.79	80.25	54.04	212.93	27	120.65	83.26	53.61	290.55	0.6133
MIP-1α	13	25.01	7.73	16.36	56.15	27	23.02	15.10	15.68	66.68	0.8624
MIP-1β	13	102.15	62.83	34.70	280.41	27	81.06	44.32	41.10	271.72	0.3334
PlGF	13	3.38	1.57	1.91	6.06	27	3.85	1.81	1.71	6.21	0.6754
SAA	13	2,683,488.00	1,218,569.00	318,141.50	13,200,000.00	27	2,681,116.00	9,488,702.00	525,220.00	164,000,000.00	0.8061
TARC	13	365.16	319.52	168.47	690.47	27	370.33	378.45	68.69	7680.34	0.7617
Tie-2	13	5847.41	2784.74	4065.41	8455.58	27	6212.88	3028.74	2440.01	10,077.48	0.7617
TNF-α	13	1.59	0.50	0.91	2.70	27	1.64	1.08	0.61	2.95	0.8966
VEGF-C	13	574.92	119.71	242.97	739.49	27	449.26	243.10	277.52	852.72	0.2919
VEGF-D	13	790.06	358.08	383.69	1126.91	27	757.22	413.20	458.68	1906.49	0.8061

Abbreviations: IQR—interquartile range; min—minimum; max—maximum; AN—anorexia nervosa; HC—healthy controls; BDNF—brain-derived neurotrophic factor; TNF—tumor necrosis factor; VEGF—vascular endothelial growth factor; IL—interleukin; VCAM-1—vascular cell adhesion molecule-1; bFGF—basic fibroblast growth factor; CRP—C-reactive protein; Flt-1—Fms-like tyrosine kinase-1; GM-CSF—granulocyte-macrophage colony-stimulating factor; ICAM-1—intercellular adhesion molecule-1, IFN-γ—interferon- γ; IP-10—interferon γ-induced protein-10; MCP—monocyte chemoattractant protein; MIP—macrophage inflammatory protein; PlGF—placental growth factor; SAA—serum amyloid A; TARC—thymus and activation-regulated chemokine; Tie-2—tyrosine kinase-2.

## Supplementary Materials

The following are available online at http://www.mdpi.com/2072-6643/10/11/1573/s1: Table S1: Findings from the linear regressions of potential confounders—age, BMI, percentage fat mass (independent variable)—on log-transformed values of inflammatory markers (dependent variable) in the whole sample; Table S2: Findings from the linear regressions of illness severity (independent variable) on log-transformed values of inflammatory markers (dependent variable) in AN patients only.
