# Supplementary material for: Inflammatory Markers in Anorexia Nervosa: An Exploratory Study
Source: Nutrients. 2018 Oct 24;10(11):1573. doi: 10.3390/nu10111573 (PMC6266841; doi:10.3390/nu10111573)
Supplement: Supplementary file 1 [file nutrients-10-01573-s001.zip › Table S1 and S2/Table S1.pdf]

Supplementary Table 1. Findings from the linear regressions of potential confounders e.g., age, BMI, percentage fat mass (independent variable), on log-transformed values of inflammatory markers (dependent variable) in the whole sample.

Inflammatory marker	Age					BMI					Percentage fat mass				
	N	df	F	*p*	% variance explained (R^2*100)	N	df	F	*p*	% variance explained (R^2*100)	N	df	F	*p*	% variance explained (R^2*100)
BDNF	40	1, 38	0.31	0.5821	0.80%	40	1, 38	**13.86**	**0.0007**	**26.47%**	39	1, 37	**7.08**	**0.0114**	**16.07%**
bFGF	40	1, 38	0.39	0.5350	1.02%	39	1, 37	0.36	0.5526	0.96%	39	1, 37	0.34	0.5651	0.90%
CRP	40	1, 38	1.68	0.2022	4.24%	40	1, 38	0.10	0.7584	0.25%	39	1, 37	0.10	0.7512	0.27%
Eotaxin	39	1, 37	1.33	0.2557	3.48%	40	1, 38	0.29	0.5906	0.77%	39	1, 37	0.33	0.5693	0.88%
Eotaxin-3	39	1, 37	**5.68**	**0.0224**	**13.32%**	39	1, 37	3.05	0.0889	7.62%	38	1, 36	1.75	0.1940	4.64%
Flt-1	37	1, 35	1.91	0.1757	5.17%	37	1, 35	0.04	0.8493	0.01%	37	1, 35	1.64	0.2090	4.47%
GM-CSF	35	1, 33	0.01	0.9163	0.03%	35	1, 33	0.78	0.3835	2.31%	33	1, 31	3.55	0.0691	10.26%
ICAM-1	40	1, 38	0.61	0.4401	1.58%	40	1, 38	0.05	0.8258	0.13%	38	1, 36	1.84	0.1834	4.86%
IFN-γ	35	1, 33	**7.91**	**0.0082**	**19.34%**	37	1, 35	2.63	0.1140	6.98%	36	1, 34	0.05	0.8294	0.14%
IL-1α	39	1, 37	0.88	0.3554	2.31%	39	1, 37	1.43	0.2394	3.72%	38	1, 36	0.03	0.8613	0.09%
IL-1β	30	1, 28	0.75	0.3932	2.62%	30	1, 28	0.73	0.4000	2.54%	30	1, 28	0.71	0.4062	2.48%
IL-2	19	1, 17	2.40	0.1397	12.37%	19	1, 17	0.00	0.9484	0.03%	18	1, 16	0.02	0.8927	0.12%
IL-4	36	1, 34	0.02	0.9012	0.05%	36	1, 34	**4.73**	**0.0367**	**12.21%**	35	1, 33	1.34	0.2545	3.92%
IL-5	35	1, 33	0.47	0.4961	1.41%	35	1, 33	2.25	0.1431	6.38%	34	1, 32	1.89	0.1791	5.57%
IL-6	40	1, 38	0.02	0.8903	0.05%	40	1, 38	**9.48**	**0.0039**	**19.96%**	39	1, 37	**8.83**	**0.0052**	**19.27%**
IL-7	40	1, 38	2.20	0.1464	5.47%	40	1, 38	0.09	0.7642	0.24%	39	1, 37	0.02	0.8794	0.06%
IL-8	40	1, 38	1.54	0.2217	3.90%	40	1, 38	0.07	0.7925	0.18%	39	1, 37	0.42	0.5200	1.13%
IL-10	39	1, 37	0.17	0.6829	0.46%	39	1, 37	**6.67**	**0.0139**	**15.26%**	38	1, 36	2.79	0.1035	7.20%
IL-12/IL-23p40	40	1, 38	0.70	0.4073	1.81%	40	1, 38	**10.97**	**0.0020**	**22.40%**	39	1, 37	**10.05**	**0.0031**	**21.37%**
IL-12p70	34	1, 32	0.16	0.6910	0.50%	34	1, 32	0.02	0.8775	0.08%	34	1, 32	0.12	0.7283	0.38%
IL-13	26	1, 24	0.51	0.4817	2.08%	26	1, 24	1.61	0.2160	6.30%	26	1, 24	1.50	0.2322	5.89%
IL-15	40	1, 38	0.27	0.6056	0.71%	40	1, 38	**9.54**	**0.0037**	**20.07%**	39	1, 37	1.42	0.2415	3.69%
IL-16	40	1, 38	0.29	0.5919	0.76%	40	1, 38	1.00	0.3245	2.55%	39	1, 37	0.42	0.5188	1.13%
IL-17A	37	1, 35	0.14	0.7148	0.39%	37	1, 35	3.97	0.0542	10.18%	36	1, 34	1.44	0.2382	4.07%
IP-10	35	1, 33	1.01	0.3233	2.96%	40	1, 38	0.98	0.3282	2.52%	39	1, 37	0.35	0.5595	0.93%
MCP-1	40	1, 38	**8.87**	**0.0050**	**18.92%**	40	1, 38	1.88	0.1784	4.71%	39	1, 37	1.17	0.2854	3.08%

MCP-4	40	1, 38	1.05	0.3127	2.68%	40	1, 38	1.30	0.2621	3.30%	39	1, 37	0.15	0.6961	0.42%
MIP-1α	40	1, 38	**4.48**	**0.0408**	**10.55%**	40	1, 38	0.07	0.7991	0.17%	39	1, 37	0.13	0.7254	0.34%
MIP-1β	40	1, 38	1.14	0.2920	2.92%	40	1, 38	2.07	0.1584	5.17%	39	1, 37	1.46	0.2347	3.79%
PlGF	40	1, 38	0.00	0.9508	0.01%	40	1, 38	0.02	0.8882	0.05%	39	1, 37	0.10	0.7581	0.26%
SAA	40	1, 38	**6.99**	**0.0119**	**15.53%**	40	1, 38	0.25	0.6226	0.64%	39	1, 37	0.35	0.5603	0.93%
TARC	38	1, 36	0.12	0.7320	0.33%	38	1, 36	0.02	0.8785	0.07%	37	1, 35	0.66	0.4214	1.86%
Tie-2	39	1, 37	0.66	0.4206	1.76%	39	1, 37	1.00	0.3230	2.64%	38	1, 36	1.77	0.1914	4.69%
TNF-α	40	1, 38	**5.07**	**0.0302**	**11.78%**	39	1, 37	0.04	0.8452	0.10%	39	1, 37	0.32	0.5730	0.87%
TNF-β	37	1, 35	1.41	0.2431	3.87%	37	1, 35	**7.45**	**0.0098**	**17.56%**	36	1, 34	**4.93**	**0.0331**	**12.67%**
VCAM-1	40	1, 38	2.10	0.1555	5.24%	40	1, 38	1.76	0.1924	4.43%	39	1, 37	2.20	0.1468	5.60%
VEGF-A	40	1, 38	0.74	0.3953	1.91%	40	1, 38	3.14	0.0763	7.63%	39	1, 37	0.91	0.3453	2.41%
VEGF-C	40	1, 38	1.18	0.2850	3.00%	39	1, 37	**6.17**	**0.0177**	**14.28%**	39	1, 37	**5.00**	**0.0315**	**11.90%**
VEGF-D	40	1, 38	0.99	0.3260	2.54%	40	1, 38	1.79	0.1890	4.50%	39	1, 37	0.13	0.7200	0.35%

*Results in bold text indicate statistical significance at $p < 0.05$.

Abbreviations: df—degrees of freedom; BMI—body mass index; BDNF—brain-derived neurotrophic factor; bFGF—basic fibroblast growth factor; CRP—C-reactive protein; Flt-1—Fms-like tyrosine kinase-1; GM-CSF—granulocyte-macrophage colony-stimulating factor; ICAM-1—intercellular adhesion molecule-1, IFN-γ—interferon- γ; IL—interleukin; IP-10—interferon γ-induced protein-10; MCP—monocyte chemoattractant protein; MIP—macrophage inflammatory protein; PlGF—placental growth factor; SAA—serum amyloid A; TARC—thymus and activation-regulated chemokine; Tie-2—tyrosine kinase-2; TNF—tumor necrosis factor; VCAM-1—vascular cell adhesion molecule-1; VEGF—vascular endothelial growth factor.
