# Supplementary material for: Inflammatory Markers in Anorexia Nervosa: An Exploratory Study
Source: Nutrients. 2018 Oct 24;10(11):1573. doi: 10.3390/nu10111573 (PMC6266841; doi:10.3390/nu10111573)
Supplement: Supplementary file 1 [file nutrients-10-01573-s001.zip › Table S1 and S2/Table S2.pdf]

Supplementary Table 2. Findings from the linear regressions of illness severity (independent variable) on log-transformed values of inflammatory markers (dependent variable) in AN patients only.

Inflammatory marker	Illness duration					Eating Disorder Symptoms					General psychopathology				
	N	df	F	*p*	% variance explained (R2*100)	N	df	F	*p*	% variance explained (R2*100)	N	df	F	*p*	% variance explained (R2*100)
BDNF	24	1, 22	0.29	0.5952	1.30%										
bFGF	24	1, 22	3.88	0.0615	15.00%	26	1, 24	3.75	0.0647	13.51%	27	1, 25	0.61	0.4427	2.38%
CRP	24	1, 22	0.09	0.7720	0.39%	27	1, 25	0.80	0.8000	3.10%	27	1, 25	0.74	0.3978	2.87%
Eotaxin	24	1, 22	0.49	0.4916	2.17%	27	1, 25	1.06	0.3124	4.08%	27	1, 25	**4.80**	**0.0380**	**16.11%**
Eotaxin-3	24	1, 22	1.43	0.2446	6.10%	27	1, 25	3.18	0.0866	11.29%	27	1, 25	4.07	0.0546	13.99%
Flt-1	23	1, 21	4.03	0.0577	16.10%	26	1, 24	1.05	0.3162	4.18%	26	1, 24	1.76	0.1968	6.84%
GM-CSF	18	1, 16	2.21	0.1566	12.13%	21	1, 19	0.00	0.9572	0.02%	21	1, 19	0.02	0.9029	0.08%
ICAM-1	24	1, 22	0.71	0.4072	3.14%	26	1, 24	2.20	0.1510	8.40%	26	1, 24	0.89	0.3538	3.59%
IFN-γ	24	1, 22	0.00	0.9518	0.02%	27	1, 25	3.87	0.0605	13.39%	24	1, 22	0.33	0.5688	1.50%
IL-1α	23	1, 21	0.17	0.6802	0.83%	26	1, 24	0.93	0.3446	3.73%	26	1, 24	0.00	0.9776	0.00%
IL-1β	20	1, 18	4.07	0.0588	18.44%	21	1, 19	0.18	0.6749	0.95%	21	1, 19	0.12	0.7375	0.61%
IL-2	12	1, 10	2.40	0.1522	19.37%	13	1, 11	0.18	0.6796	1.61%	13	1, 11	0.05	0.8219	0.48%
IL-4	20	1, 18	**15.82**	**0.0009**	**46.77%**	23	1, 21	0.15	0.7068	0.69%	23	1, 21	0.21	0.6541	0.97%
IL-5	21	1, 19	0.00	0.9586	0.01%	23	1, 21	0.58	0.4538	2.70%	23	1, 21	1.47	0.2389	6.54%
IL-6	24	1, 22	0.79	0.3828	3.48%	27	1, 25	1.43	0.2436	5.40%	27	1, 25	0.01	0.9130	0.05%
IL-7	24	1, 22	0.32	0.5794	1.42%	27	1, 25	1.33	0.2594	5.06%	27	1, 25	**4.51**	**0.0439**	**15.27%**
IL-8	24	1, 22	1.47	0.2386	6.25%	27	1, 25	1.22	0.2801	4.65%	27	1, 25	**9.88**	**0.0043**	**28.32%**
IL-10	23	1, 21	2.23	0.1500	9.61%	26	1, 24	4.02	0.0563	14.36%	26	1, 24	0.32	0.5742	1.33%
IL-12/IL-23p40	23	1, 21	**6.70**	**0.0172**	**24.19%**	27	1, 25	2.15	0.1554	7.91%	27	1, 25	0.00	0.9833	0.00%
IL-12p70	22	1, 20	1.35	0.2595	6.31%	24	1, 22	0.00	0.9723	0.01%	24	1, 22	0.03	0.8691	0.13%
IL-13	14	1, 12	0.70	0.4201	5.49%	17	1, 15	1.99	0.1793	11.69%	17	1, 15	0.71	0.4119	4.53%

IL-15	23	1, 21	0.38	0.5446	1.77%	26	1, 24	3.81	0.0628	13.69%	27	1, 25	0.50	0.4874	1.95%
IL-16	24	1, 22	2.29	0.1445	9.43%	27	1, 25	1.82	0.1897	6.78%	27	1, 25	3.60	0.0695	12.58%
IL-17A	22	1, 20	2.28	0.1463	10.25%	25	1, 23	0.31	0.5831	1.33%	25	1, 23	0.02	0.8796	0.10%
IP-10	23	1, 21	2.27	0.1469	9.75%	25	1, 23	**12.60**	**0.0017**	**35.40%**	27	1, 25	**6.10**	**0.0207**	**19.62%**
MCP-1	23	1, 21	**6.40**	**0.0194**	**23.37%**	27	1, 25	0.48	0.4952	1.88%	27	1, 25	**5.52**	**0.0270**	**18.08%**
MCP-4	24	1, 22	0.70	0.4110	3.09%	27	1, 25	0.05	0.8218	0.21%	27	1, 25	0.29	0.5967	1.14%
MIP-1α	24	1, 22	4.12	0.0548	15.76%	27	1, 25	0.80	0.3811	3.08%	27	1, 25	1.42	0.2443	5.38%
MIP-1β	23	1, 21	0.00	0.9976	0.00%	26	1, 24	1.49	0.2339	5.85%	26	1, 24	0.01	0.9299	0.03%
PlGF	24	1, 22	0.01	0.9284	0.04%	27	1, 25	**4.44**	**0.0454**	**15.07%**	27	1, 25	2.88	0.1019	10.34%
SAA	24	1, 22	1.84	0.1882	7.73%	27	1, 25	3.27	0.0827	11.56%	27	1, 25	1.67	0.2082	6.26%
TARC	23	1, 21	0.55	0.4662	2.56%	25	1, 23	3.59	0.0709	13.49%	24	1, 22	**9.18**	**0.0062**	**29.44%**
Tie-2	23	1, 21	0.53	0.4740	2.47%	26	1, 24	1.20	0.2849	4.75%	26	1, 24	1.93	0.1781	7.43%
TNF-α	24	1, 22	3.09	0.0926	12.32%	27	1, 25	0.49	0.4903	1.92%	26	1, 24	0.01	0.9373	0.03%
TNF-β	22	1, 20	0.26	0.6170	1.27%	26	1, 24	0.85	0.3667	3.41%	26	1, 24	3.52	0.0730	12.78%
VCAM-1	24	1, 22	2.01	0.1706	8.36%	27	1, 25	0.18	0.6739	0.72%	27	1, 25	0.83	0.3703	3.22%
VEGF-A	24	1, 22	**5.55**	**0.0278**	**20.15%**	27	1, 25	0.44	0.5119	1.74%	27	1, 25	0.08	0.7758	0.33%
VEGF-C	24	1, 22	0.00	0.9459	0.02%	27	1, 25	2.91	0.1006	10.42%	27	1, 25	1.83	0.1886	6.81%
VEGF-D	24	1, 22	2.59	0.1220	10.52%	27	1, 25	0.00	0.9805	0.00%	27	1, 25	1.89	0.1812	7.03%

*Results in bold text indicate statistical significance at $p < 0.05$.

Abbreviations: df—degrees of freedom; BDNF—brain-derived neurotrophic factor; bFGF—basic fibroblast growth factor; CRP—C-reactive protein; Flt-1—Fms-like tyrosine kinase-1; GM-CSF—granulocyte-macrophage colony-stimulating factor; ICAM-1—intercellular adhesion molecule-1, IFN-γ—interferon- γ; IL—interleukin; IP-10—interferon γ-induced protein-10; MCP—monocyte chemoattractant protein; MIP—macrophage inflammatory protein; PlGF—placental growth factor; SAA—serum amyloid A; TARC—thymus and activation-regulated chemokine; Tie-2—tyrosine kinase-2; TNF—tumor necrosis factor; VCAM-1—vascular cell adhesion molecule-1; VEGF—vascular endothelial growth factor.
